# Alcohol, Aldehydes, Adducts and Airways

**DOI:** 10.3390/biom5042987

**Published:** 2015-11-05

**Authors:** Muna Sapkota, Todd A. Wyatt

**Affiliations:** 1 Department of Environmental, Agricultural and Occupational Health, College of Public Health, University of Nebraska Medical Center, Omaha, NE 68198, USA; E-Mail: muna.sapkota@unmc.edu; 2 Department of Internal Medicine, Division of Pulmonary, Critical Care, Sleep and Allergy, University of Nebraska Medical Center, Omaha, NE 68198, USA; 3 VA Nebraska-Western Iowa Health Care System, Omaha, NE 68105, USA

**Keywords:** lipid peroxidation, reactive oxygen species, alcohol, cigarette smoke, aldehydes, adducts, lung

## Abstract

Drinking alcohol and smoking cigarettes results in the formation of reactive aldehydes in the lung, which are capable of forming adducts with several proteins and DNA. Acetaldehyde and malondialdehyde are the major aldehydes generated in high levels in the lung of subjects with alcohol use disorder who smoke cigarettes. In addition to the above aldehydes, several other aldehydes like 4-hydroxynonenal, formaldehyde and acrolein are also detected in the lung due to exposure to toxic gases, vapors and chemicals. These aldehydes react with nucleophilic targets in cells such as DNA, lipids and proteins to form both stable and unstable adducts. This adduction may disturb cellular functions as well as damage proteins, nucleic acids and lipids. Among several adducts formed in the lung, malondialdehyde DNA (MDA-DNA) adduct and hybrid malondialdehyde-acetaldehyde (MAA) protein adducts have been shown to initiate several pathological conditions in the lung. MDA-DNA adducts are pre-mutagenic in mammalian cells and induce frame shift and base-pair substitution mutations, whereas MAA protein adducts have been shown to induce inflammation and inhibit wound healing. This review provides an insight into different reactive aldehyde adducts and their role in the pathogenesis of lung disease.

## 1. Introduction

The lung is a highly specialized organ charged with the principal role of O_2_/CO_2_ exchange between atmosphere and bloodstream [1]. In addition to this gas exchange, it also serves as an interface between host and external environment [1]. In this regard, the lungs can be considered an external organ due to continual exposure to ambient air [2]. The enormous surface area of the airways and continuous exposure to external air makes the lung vulnerable to numerous inhaled toxicants, gases, pathogens and chemicals [2]. All of these exposures make the lung susceptible to varying degrees of physical, chemical, and biological insults [3]. To combat these insults and to defend against inhaled pathogens and other toxicants, the lung employs a defense mechanism including exhalation, cough reflex, ciliary beat, and mucus clearance [4], as well as a highly complex innate immune system including airway epithelial cells [5] and resident and recruited leukocytes [2]. This first line of defense is later followed by specific acquired immune responses associated with the activation of T and B cells aimed against specific antigens [6]. In the case of continuous insults, lung defense is compromised, allowing inhaled toxic agents to stimulate the generation of reactive oxygen species (ROS) [7]. These ROS induce intracellular responses resulting in the release of pro-inflammatory cytokines and chemokines [7] that stimulate the influx of neutrophils and monocytes into the lung [7]. Continuous inhalation of pathogens or toxic agents, however, may result in excessive ROS production, leading to chronic inflammation and lung injury [8]. If not controlled, these ROS may induce inflammation and DNA damage, inhibit apoptosis, and may also activate proto-oncogenes through initiation of several signal transduction pathways [9]. Therefore, oxidative stress associated with increased production of ROS in the lung due to various toxic inhalants may predispose individuals to lung diseases such as chronic obstructive pulmonary disease (COPD) [10].

## 2. Role of Alcohol in Lung Disease

Chronic alcohol abuse or alcoholism costs about $223 billion annually to the U.S. economy [11] and is the fourth leading preventable cause of death, causing more than 88,000 deaths annually [12]. Drinking more than two drinks/day for men and one drink/day for women can have deleterious health effects and is associated with increased mortality [13]. Alcohol abuse is common among critically ill patients and is attributed to about 40% of admissions to the intensive care unit [14]. Tissue injury to liver, stomach and brain as well as cancers of the upper aero-digestive tract, stomach, and liver are known health risks associated with chronic alcohol consumption [15]. In the lung, alcohol over-consumption predisposes the host to infectious diseases such as pneumonia [16], as well as acute respiratory distress syndrome (ARDS) [17]. After oral ingestion, less than 10% of alcohol consumed is excreted unchanged in urine, sweat and breath [18,19]. In the lung, due to alcohol’s volatility, it diffuses freely from the bronchial circulation into the airways, rapidly condenses with decreasing temperature, and deposits back onto the airways. This recycling of alcohol vapor (or “rain effect”) potentially results in a high concentration of alcohol in the airways [20]. Thus, exhaled alcohol breath tests are commonly used to measure alcohol ingestion by law enforcement agencies in estimating blood alcohol levels [21].

Bacterial infection and acute lung injury are the most significant pulmonary effects of such alcohol abuse [22]. Increased risk for infection with tissue-damaging gram-negative pathogens, such as *Klebsiella pneumonia*, is common in alcoholic patients [23]. Other risks associated with alcohol abuse are aspiration of gastric acid and/or microbes and impairment of mucous-facilitated clearance of bacterial pathogens [22]. In part, this explains the increased risk of respiratory infections in individuals with alcohol use disorders (AUDs). Alcohol-mediated suppression of host immune response and pathogen-clearing function of alveolar macrophages could further explain the increased risk of both bacterial pneumonia and tuberculosis [24].

Production of white blood cells in the bone marrow and superoxide production in neutrophils are also decreased in chronic alcohol consumption [25]. Chronic alcohol consumption increases alveolar capillary permeability, protein concentration in the alveolar lining fluid and pulmonary edema formation in lung [26]. Chronic alcohol ingestion also depletes the antioxidant, glutathione (GSH), throughout the alveolar lining fluid of the lung and within macrophages [17]. Other deleterious effects include abnormal synthesis and secretion of lung surfactants and increased apoptosis of type II cells [27].

Although alcohol has many adverse effects on lung function, only a limited number of studies have examined the biochemical processes involved in the mechanism of such injury. Interaction of alcohol’s metabolites with other exposures could be one of several possible causes [28]. Therefore, development of alcohol co-exposure markers in the lung could be of interest in understanding the pathogenesis of lung disorders associated with alcohol abuse.

## 3. Role of Cigarette Smoking in Lung Disease

Cigarette smoking is the number one preventable cause of death in the United States, resulting in 480,000 deaths each year [29]. A causal association between cigarette smoking and cancers of lung, liver, nasopharynx, oropharynx, and larynx has been established by epidemiological studies [30,31,32]. In developed countries, cigarette smoking attributes to approximately 90% of lung cancer cases in males and 80% in females [33,34]. The number of cigarettes smoked, inhalation practice, duration and early start of smoking are the critical risk factors [35]. Thousands of chemicals contained in tobacco smoke are known to have carcinogenic properties and can undergo metabolic activation in tissue leading to formation of reactive intermediates [36,37]. Besides being a risk factor for cancer development, smoking is also the main cause for COPD development [38].

Cigarette smoke contains high concentrations of free radicals in both the gas and tar phases [35]. These stable oxidized intermediates induce endogenous oxidative stress and inflammation [39]. Oxidative DNA damage and lipid peroxidation (LPO) of cell membranes are important effects of cigarette smoke-induced oxidative injury [40]. LPO provides a continuous supply of free radicals for the oxidation of polyunsaturated fatty acids in membranes causing oxidative cell damage [41]. Cigarette smoke-mediated oxidative stress induces local inflammation resulting in increased numbers of macrophages in the lung [42]. These macrophages recruit additional inflammatory cells into the lung including neutrophils, monocytes, eosinophils, and T lymphocytes [43]. The result is a destructive cascade of exposure of the elastolytic compounds and ROS that destroy the lung structure resulting in emphysema and obstructive bronchitis [44]. In addition, high carbonyls content such as acrolein and 4-hydroxynonenal (4-HNE) in the cigarette smoke also leads to carbonyl stress in the lung [45]. Other carbonyl compounds present in cigarette smoke are formaldehyde, acetaldehyde, propanal and malondialdehyde [46]. These carbonyls generated as a result of oxidative stress may play an important role in the progression of lung disease such as COPD [47].

## 4. Other Environmental Oxidants

In recent years, ambient air pollutants and diesel exhaust particles have been linked to oxidative damage in cells and in tissue [48]. Air pollutants also contribute to oxidative stress in the pulmonary system and play a role in adverse lung effects [49]. Ozone, a secondary air pollutant, is a known pulmonary irritant [50]. In addition to a single agent, exposure to combined air pollutants, such as ozone and particulate matter (PM), greatly induces pulmonary oxidative stress and inflammation [50]. This could explain the association between environmental air pollutants and increase in pulmonary diseases and mortality demonstrated by several clinical and epidemiological studies [51,52,53].

## 5. Source of Aldehydes in the Lung

**Figure 1:**
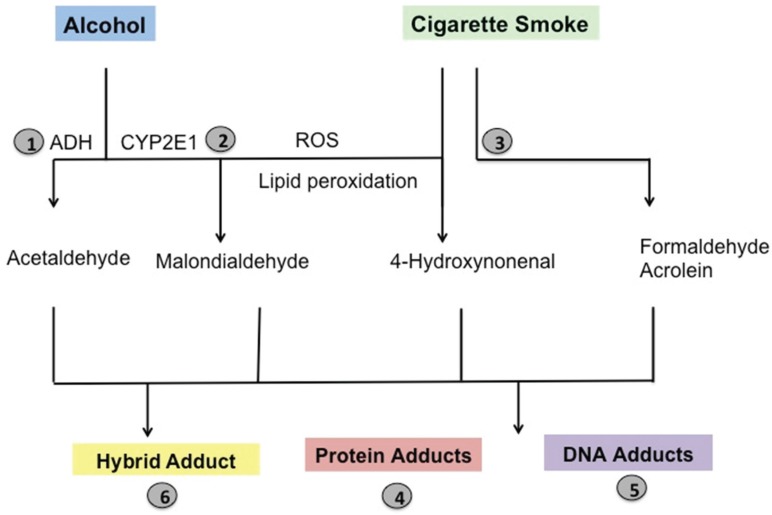
Generation of lung aldehydes and adduct formation. Alcohol is metabolized by alcohol dehydrogenase (ADH) to acetaldehyde (AA). But during chronic alcohol consumption, CYP2E1 is induced leading to generation of ROS like superoxide, hydrogen radical and hydrogen peroxide. This promotes lipid peroxidation and generation of malondialdehyde (MDA) and 4-hydroxynonenal (4-HNE). Cigarette smoke itself contains high concentration of AA, acrolein and formaldehyde. In addition to this, smoking cigarettes also induces local inflammation in lung causing more generation of ROS. This further promotes lipid peroxidation generating more MDA and 4-HNE. Acetaldehyde and MDA could form hybrid adduct through Schiff base reaction when 2 mole of MDA react with 1 mole of AA to form a stable hybrid adduct [74,75,76,77].

Significant amounts of ingested alcohol reach the airways via the bronchial circulation where it is either metabolized or is excreted by exhaling the vapor [54]. Although the majority of ingested alcohol is metabolized in the liver, the mammalian lung can also metabolize ingested alcohol through the action of alcohol dehydrogenase (ADH) to acetaldehyde [54]. Thus, after alcohol consumption, airways are exposed to high concentrations of acetaldehyde, a primary metabolite (Figure 1) [55]. In addition to ADH, during chronic alcohol consumption, alcohol is metabolized by microsomal cytochrome P450 2E1 (CYP2E1) and peroxisomes to generate ROS leading to oxidative stress [56]. Human lung cells, especially bronchial epithelium, club cells, type II pneumocytes, and alveolar macrophages, have been shown to express CYP enzymes [57]. CYP2E1-generated ROS easily react with lipid membranes causing LPO [58], which is important in the generation of reactive aldehydes such as malondialdehyde (MDA) and other products, like 4-HNE [59,60]. 4-HNE forms Michael adducts with nucleophilic sites in DNA, lipids and proteins [60]. Another major source of reactive aldehydes in the lung is from the vapor phase of cigarette smoke, which is known to contain several aldehydes including butyraldehyde, isobutyraldehyde, propionaldehyde, and acetaldehyde [61]. Among the different aldehydes contained in cigarette smoke, acetaldehyde is the major one, presenting in very high concentrations [62] (approximately 920 μg per cigarette) [63]. Additionally, acetaldehyde is widely used as a natural constituent of foods and is present in the environment as a pyrolysis product [64]. Acetaldehyde and MDA also are produced in biologically significant amounts during the metabolism of alcohol [65]. Higher levels of aldehydes have also been reported in exhaled breath condensate and saliva in current smokers and patients with COPD [66,67]. Aldehydes have also been identified in the bronchoalveolar lavage (BAL) fluid of animals exposed to ozone [68]. These aldehydes, especially acrolein, MDA, formaldehyde and crotonaldehyde, are highly reactive and could form DNA adducts in a variety of human tissues [69]. Additionally, significantly elevated levels of DNA adducts and smoking-related protein adducts were detected in BAL cells as well as in the bronchial epithelium and the peripheral lung of smokers [70,71]. The lung is also vulnerable to oxidative injury as a result of exercise and high altitude exposure due to oxidative stress [72]. In addition to the lung, increased MDA levels are also reported in excreted urine of patients with COPD after exercise as a result of exercise-induced stress [73].

## 6. Pathological Implications of Lung Aldehydes

The accumulation of LPO products in human tissues is a major cause of cellular and tissue dysfunction as it may lead to membrane dysfunction and oxidative stress-related diseases [78]. Reactive α, β-unsaturated aldehydes generated as a result of LPO could contribute to vascular disease and other oxidative stress-related pathologies due to modification of biomolecules [79]. Because oxidative stress plays an important role in the development and/or progression of vascular diseases such as atherosclerosis, serum malondialdehyde and malondialdehyde-acetaldehyde levels are used as biological markers of oxidative stress [80]. 4-HNE, a highly reactive end product of LPO [10], has been linked to a number of pathologies such as alcoholic liver disease, COPD, emphysema, asthma, Alzheimer’s disease and Parkinson’s disease [81]. In the lung during oxidative stress, reactive LPO products are degraded very slowly, resulting in greater accumulation of these products leading to extensive adduct formation and tissue damage [60]. Acetaldehyde may also trigger asthma attacks in individuals with genetic alcohol dehydrogenase polymorphisms [82]. Inhalation of acetaldehyde for 30 min causes mild respiratory irritation [83]. Acetaldehyde also has a significant role in the etiology of lung cancer [59]. Aldehydes in cigarette smoke are able to induce the pro-inflammatory cytokines, tumor necrosis factor alpha (TNF-α) and interleukin-6 (IL-6) from macrophages, and the neutrophil chemokine, interleukin-8 (IL-8), from human bronchial epithelial cells [84,85]. Aldehydes contained in cigarette smoke have been shown to inhibit human neutrophil apoptosis and contribute to neutrophilic accumulation, resulting in the delayed resolution of inflammation [86]. Acrolein, one of the major constituents of cigarette smoke, is involved in increased mucin production and regulation of lung matrix metalloproteinase 9 (MMP-9), which may result in decreased lung function in COPD patients [87]. In addition to this, glutathione is irreversibly modified by acrolein and crotonaldehyde in human airway epithelial cells [88]. Acetaldehyde is also considered a toxin with epigenetic and genetic effects [89]. Ethanol-induced hepatic steatosis, fibrosis, carcinoma and gastrointestinal injury are attributed to alcohol-mediated oxidative stress [90]. Lipid peroxidation affects mitochondrial membrane permeability [91]. Similarly, acetaldehyde could also inhibit mitochondrial reactions and functions [91]. Acrolein may have a role in epigenetic modification as it is known to form adducts with histone protein [92]. In brief, in addition to oxidative stress and immune dysfunction, membrane disruption, histone modification and mitochondrial dysfunction are other major pathological implications of aldehydes (Table 1).

**Table 1:** Lung aldehydes, type of adducts formed and lung effect.

**Lung Aldehydes**	**Source**	**Lung Effect**
Acetaldehyde		Oxidative stress [78,79]
Malondialdehyde	Alcohol	COPD, asthma, emphysema [81,82]
4-Hydroxynonenal	Cigarette smoke	Mild respiratory irritation [83]
Acrolein	Environmental toxicants	Release of pro-inflammatory cytokine [84,85]
Formaldehyde		Epigenetic and genetic effect [89]
**Lung Adducts**	**Aldehydes Involved**	**Lung Effect**
Protein adduct		Damage protein structure and function [93,94]
	Acetaldehyde,	Slow cilia beating [95]
	Malondialdehyde,	Inhibition of anti-oxidative defense [96]
	4-hydroxynonenal	Stimulation of fibrogenesis [97,98]
		and induction of immune response [99,100,101]
DNA adduct	Acetaldehyde,	Base pair mutation [102,103]
	Malondialdehyde,	Carcinogenesis [104,105]
	Formaldehyde	Increased risk of mutation [102,106]
Hybrid adduct	Acetaldehyde, Malondialdehyde	Induce pro-inflammatory chemokine [107]
		Inhibit bronchial epithelial cell wound closure [108]
		Increase influx of neutrophils [74]

Even though chronic alcohol ingestion and cigarette smoke are two major sources of aldehydes in the lung, few studies have been conducted on the co-exposure of alcohol and cigarette smoke in the lung. This co-exposure is important because the highest level of aldehydes is generated when lungs are co-exposed to cigarette smoke and alcohol [54,109]. This co-exposure often leads to oxidative stress resulting in high concentrations of acetaldehyde and malondialdehyde in the lung [109]. Therefore, the reactive aldehydes generated in the lung could be related to various lung pathologies associated with alcohol abuse and cigarette smoking.

## 7. Lung Aldehydes and Protein Adduction

**Figure 2:**
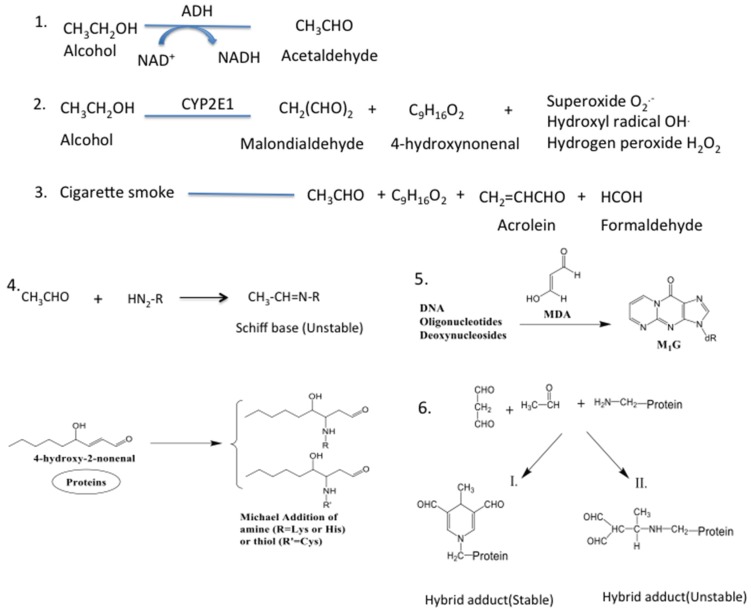
Acetaldehyde and MDA could form hybrid adduct through Schiff base reaction when 2 mole of MDA react with 1 mole of AA to form a stable hybrid adduct. Aldehydes like AA, MDA, acrolein, 4-HNE and formaldehyde could also form protein adduct and DNA adduct. The main reactions involved are Schiff base (involves binding of aldehyde to the alpha group of an N-terminal amino acid of the protein) and Michael addition (involves of binding of aldehyde on amino groups (Lys and His) or thiols (Cys or GSH) [74,75,76,77].

Reactive aldehydes are electrophilic and react with a nucleophilic site that donates an electron to form a strong covalent bond leading to adduct formation [110]. Generally, one of two chemical reactions are involved in adduct formation (Figure 2) [111]. One is a Michael addition, which is the reaction between β-carbon of aldehydes and nucleophilic groups to form 1,2-addition with the double bond. Secondly, there is a base reaction that involves formation of Schiff bases between the carbonyl carbon of aldehyde and the primary amino group of lysine or N-terminal residues [112]. Various stable and unstable adducts are formed when reactive aldehydes generated covalently bind to amino acid residues of proteins [113]. Such adduction may disturb protein cellular functions [93]. Aldehydes damage protein structure by forming adducts through covalent bonding with cysteine, lysine or histidine residues [94]. In the lung, both ADH- and CYP2E1-catalyzed metabolism of alcohol is associated with generation of acetaldehyde, a reactive aldehyde capable of binding to cellular proteins [58]. Acetaldehyde, the first metabolite of alcohol, is highly reactive and forms adducts primarily by binding to reactive lysine residues of preferred target proteins [114]. Acetaldehyde also has the ability to covalently bind to several proteins that could be detrimental to the protein function [115], due to formation of both stable and unstable adducts with various proteins [116]. In addition, other lipid peroxidation-generated aldehydic products such as MDA and HNE also form Schiff base adducts with proteins [117].

Several proteins such as albumin, tubulin, lipoproteins, collagens and erythrocyte membrane proteins serve as targets for aldehyde adduction [118]. Accumulation of acetaldehyde due to excess alcohol consumption can lead to increased interaction of this aldehyde with biomolecules [28]. Tobacco smoke is another source of oxidative stress in the lung as it induces the production of aldehyde-mediated injury through oxidative DNA damage and lipid peroxidation of cell membranes [119]. In addition to acetaldehyde, 4-HNE has been known to form protein adducts with insulin and histidine residues in proteins [120].

## 8. Pathological Implications of Protein Adducts

Slow cilia beating and decreases in cilia dynein ATPase activity have been reported as a result of acetaldehyde binding with dynein and tubulin proteins important for cilia motion [95]. Adduction of acetaldehyde with GSH inhibits the anti-oxidative defense system (AODS) responsible for the detoxification of ROS and reactive nitrogen species (RNS) [96]. Stimulation of nuclear factor-kappa B (NFκB), which regulates the secretion of pro-inflammatory cytokines, is another effect of acetaldehyde [121]. Aldehyde products stimulates fibro genesis by increasing the expression of connective tissue proteins and extracellular matrix components [97,98] and induces immune responses [99,100,101]. Also, 4-HNE formed during lipid peroxidation after ozone exposure appears to form specific protein adducts which is toxic and cause apoptosis of murine lung cells [122]. Acute alcohol toxicity may lead to formation of malondialdehyde protein adduct in the muscle [123]. The presence of HNE-protein adducts has also been studied in diseases related to oxidative stress such as neurodegenerative diseases and atherosclerosis [124]. Similarly, protein adducts of acrolein may have a role Alzheimer’s disease, Parkinson’s disease [125], atherosclerosis [126] and chronic obstructive lung disease [127]. Exposure of 4-HNE to THP-1 cells resulted in modification of proteins and enzymes involved in cytoskeleton organization, stress responses, and other metabolic pathways [128]. MDA and 4-HNE protein adduct formation in the liver could play an important role in the development and progression of alcoholic liver disease [129,130]. In COPD patients, a large number of carbonyl-modified proteins has been reported in the peripheral lung tissue and correlated with disease severity measured by the decline in forced expiratory volume in 1 second (FEV_1_) [131]. Aldehyde-modified protein formation also has an effect on cellular responses. 4-HNE adduction with extracellular signal-regulated kinases (ERK1/2) decreased ERK-1/2 phosphorylation and nuclear localization [132]. Similarly, the modification of adenosine monophosphate-activated protein (AMP) kinase with 4-HNE inhibits its kinase activity and attenuates downstream AMP kinase signaling pathway in MCF-7 breast cancer cells [133]. HNE forms adducts with c-Jun amino-terminal kinases (JNKs) leading to nuclear translocation and activation in human hepatic stellate cells [134]. Aldehyde adduct also interferes with the function of extracellular matrix protein, which could lead to the formation of scar tissue in the liver [65]. MDA, and 4-HNE modified proteins has also been studied in human eye disease [135].

## 9. Lung Aldehydes and DNA Adduction

Acetaldehyde is highly reactive and the electrophilic nature of its carbonyl carbon results in reactions with DNA, generating DNA adducts [136]. This could explain the cytotoxic, genotoxic, mutagenic, and clastogenic nature of acetaldehyde as DNA adduct formation plays a critical role in carcinogenesis [104,105]. Most of these effects have been proposed to originate from a variety of DNA-acetaldehyde adducts [104]. DNA base deoxyguanosine (dG) is the major target for adduction followed by deoxyadenosine (dA) and then deoxycytosine (dC) [137]. Acetaldehyde forms other DNA adducts such as N2-ethyl-2'-deoxyguanosine (N2-Et-dG) [138] and 1,N2-propano-2'-deoxyguanosine (PDG) [139]. Another well-studied aldehyde-DNA adduct is the crotonaldehyde-derived propano-dG (CrPdGs) adduct [136]. MDA, a natural product of lipid peroxidation, is also capable of forming an exocyclic DNA adduct named malondialdehyde-deoxyguanosine adduct (M1dG) after its interaction with DNA [140]. MDA-DNA adduct is also formed when base propenal intermediate is formed during direct DNA oxidation [141]. 4-HNE, a well-known end product of LPO, also forms exocyclic ethanol DNA adducts, which are highly carcinogenic [142]. Formaldehyde, an aldehyde contained in cigarette smoke, also induces ROS formation in many tissues, which can further interact with DNA [143] Malondialdehyde formed in the lung of cigarette smokers could form adducts with DNA bases and may damage such macromolecules [39].

## 10. Pathological Implications of DNA Adducts

DNA damage is one of the important pathological conditions associated with DNA adduct formation as this could increase the risk of somatic mutations [106] by inducing base pair mutations and causing frame-shift mutations [102,103]. MDA-DNA adducts in a number of tissues, including liver [144], breast [145] and oral mucosal cells [146]. Another DNA adduct, M1dG, may be associated with increased cancer risk and tumor progression [106]. MDA-DNA adducts might contribute to the cause of tobacco-related laryngeal cancer as these adducts have been detected in the bronchial epithelium and in the larynx of smokers [39,145]. Also, an increased level of MDA-dA was reported in the larynx of subjects with the highest intake of alcohol (>44 g) [147]. M1dG adduct was also detected both in human bronchial epithelial cells and mouse lung tissue exposed to alcohol [148]. In addition to lung, MDA-DNA adducts were also detected in tissues from patients with breast cancer [145]. A correlation was found between CYP2E1, 4-HNE and exocyclic ethanol adducts of adenine and cytosine in patients with alcoholic liver disease [149]. M1dG adducts were also detected on leukocytes exposed to formaldehyde [140] and industrial air pollution [106]. DNA adducts formed by acetaldehydes could prompt replication errors and mutations in oncogenes or onco-suppressor genes, which increases risk for carcinogenesis [150].

## 11. Lung Aldehydes and Hybrid Adducts

People with AUDs are two to three times more likely to smoke cigarettes than those without AUDs [151]. This suggests more frequent and higher rates of cigarette smoking among those with AUDs than in the general population [152]. Also a strong correlation exists between alcohol and tobacco consumption and heavy drinkers have more trouble quitting smoking than do light drinkers [153]. In the lung, a unique aldehyde environment is created during co-exposure to alcohol and cigarette smoke due to the generation of a high concentration of aldehydes [74]. For instance, high concentrations of acetaldehyde and malondialdehyde were detected in the BAL fluid of mice co-exposed to cigarette smoke and alcohol [109]. This elevated level of aldehydes is of importance as it is necessary for the formation of the hybrid malondialdehyde-acetaldehyde (MAA) adduct in mouse lung [109]. Formation of five different types of protein adducts, acetaldehyde, MDA, MAA, 4-HNE and hydroxyl ethyl radical, are reported to form after ethanol consumption [154]. MAA-adducted proteins are highly stable and resistant to rapid degradation [109]. This hybrid adduct is composed of a cyclic product consisting of two molecules of MDA and one molecule of acetaldehyde as a result of a Schiff base reaction described previously [155]. The MAA adduct is highly fluorescent and can be detected for a few weeks in the liver as a result of slow degradation [156,157]. In comparison to single exposure to alcohol or smoke *alone*, MAA adducts have been detected only in the lungs of mice exposed to *both* alcohol and cigarette smoke [109]. Different endogenously nucleophilic proteins contained in the lung are the target of MAA to form hybrid adducts [109]. Among these, surfactant protein A (SP-A) and surfactant protein D (SP-D) synthesized primarily by type II alveolar cell in the alveolus have been extensively studied [74,158]. SP-A and SP-D play an important role in innate immunity as they can directly kill bacteria, or can act as an opsonizing agent by binding to bacteria subsequently enhancing macrophage phagocytosis [159]. MAA-adducted proteins are good ligands for scavenger receptor A (SRA; CD204), which are expressed extensively on macrophages and also found on endothelial cells, platelets, and epithelial cells [160,161]. MAA stimulates inflammatory responses in airway epithelial cells through binding to SRA [158]. Diminished antibody responses to MAA-bovine serum albumin (MAA-Alb) in SRA knockout mice have also been previously reported [160]. In addition, pre-treatment with SRA-binding ligand, fucoidan, blocked MAA adduct-mediated release of pro-inflammatory chemokine IL-8 [107].

## 12. Pathological Implications of Hybrid Adduct

The hybrid adduct, MAA, has been reported to induce pro-inflammatory responses and delay wound healing in airway epithelial cells. MAA adduct stimulates release of the neutrophil chemokine, IL-8, when exposed to bronchial epithelial cells [107]. Similarly, intranasal instillation of SPD-MAA in mice induced KC (CXCL1), a homolog of human IL-8, in comparison to saline or non-adducted SPD control [74]. This elevation in KC release resulted in an influx of neutrophils in the lungs of mice instilled with MAA adduct for 3 weeks [74]. MAA adduct-stimulated cytokine release is blocked by protein kinase C (PKC) inhibitors, implicating a role for PKC in MAA-adducted protein-stimulated IL-8 release from bronchial epithelial cells [107]. MAA adducts have also been shown to inhibit bronchial epithelial cell wound closure [108]. MAA adduct-induced inflammation is also mediated through PKC as MAA adducts activate PKC epsilon in tracheal epithelial cells [74,107]. Immunologic reactions associated with alcohol-related liver disease and atherosclerosis-induced vascular inflammatory injury also have been associated with MAA adduct formation [162,163]. In addition to IL-8, MAA adducts have been reported to induce the expression of inflammatory cytokines such as TNF, intracellular adhesion molecule and vascular cell adhesion molecule in liver endothelial cells [164]. Increased formation of MAA adduct has also been reported in rheumatoid arthritis synovial tissue [165]. MAA adducts also induce an antibody response as T helper and cytotoxic T cells exhibit robust antibody responses to MAA epitope [166]. Extent of tissue damage in acute injury and chronic disease states such as atherosclerosis could be correlated to this antibody response [163]. Circulating MAA-modified proteins in the bloodstream could be bound, internalized, degraded and presented to the cells of the immune system resulting in an immune response [167]. Formation of MAA adducts with N-terminal and bait region of mouse alpha 2 macroglobulin (A2M) has been shown to modulate its proteinase and TGF-b1 binding function [168].

## 13. Conclusions

Chronic alcohol consumption and cigarette smoking result in the production of several types of aldehyde adducts in the lung. The formation of these adducts leads to impaired function and induces inflammation and mutagenesis. Although chronic alcohol abuse predisposes the host to pneumonia and ARDS, very few studies have focused on the role of alcohol metabolism in alcohol-induced toxicity and its consequences in the lung. Many studies have been directed toward cigarette smoke-induced oxidative stress, but it has been shown that alcohol also increases LPO leading to the generation of reactive aldehydes such as acetaldehyde and MDA. Because the lung is continuously exposed to high concentration of alcohol in heavy drinkers, alcohol significantly contributes to the high level of aldehydes detected in the lung. Several mechanisms have been proposed to understand the consequences of alcohol-induced liver injury, but only limited studies have been done in the case of the lung. Additional studies are required to further clarify the role of alcohol in oxidative stress and aldehyde generation in the lung. Additional studies are needed to determine the role of different adducts formed in the lung and their role in lung pathogenesis. As ROS-mediated lipid peroxidation is a major source of aldehyde generation in lung, it is also important to study different factors that stimulate ROS generation. Different reactive aldehydes and adducts formed in the lung could act as potential biological markers for the source and degree of lung injury associated with alcohol, cigarette smoke and other inhaled environment pollutants. Discovering innovative approaches to better identify the mechanisms through which adducts cause lung injury, however, still remains a challenge for researchers. Among all adducts, the stability of the MAA hybrid adduct may play a prominent role in mediating the long term consequences of chronic alcohol abuse and cigarette smoking with respect to the development of respiratory infections as well as emphysema and COPD. Understanding factors regulating adduct production and their role in the progression of chronic lung diseases is necessary and important in order to develop new therapeutic approaches targeting the formation and accumulation of reactive aldehyde adducts for promoting the resolution of lung injury.
